# Comparative population genetics of the invasive mosquito *Aedes albopictus* and the native mosquito *Aedes flavopictus* in the Korean peninsula

**DOI:** 10.1186/s13071-021-04873-5

**Published:** 2021-07-27

**Authors:** Jiyeong Shin, Jongwoo Jung

**Affiliations:** 1 The Division of EcoCreative, Ewha Womans University, Seoul, 03760 South Korea; 2 Department of Science Education, Ewha Womans University, Seoul, 03760 South Korea

**Keywords:** *Aedes albopictus*, *Aedes flavopictus*, Population structure, Genetic diversity

## Abstract

**Background:**

*Aedes* mosquitoes are important invasive species contributing to the spread of chikungunya, dengue fever, yellow fever, zika virus, and other dangerous vector-borne diseases. *Aedes albopictus* is native to southeast Asia, with rapid expansion due to human activity, showing a wide distribution in the Korean peninsula. *Aedes flavopictus* is considered to be native to East Asia, with a broad distribution in the region, including the Korean peninsula. A better understanding of the genetic diversity of these species is critical for establishing strategies for disease prevention and vector control.

**Methods:**

We obtained DNA from 148 specimens of *Ae. albopictus* and 166 specimens of *Ae. flavopictus* in Korea, and amplified two mitochondrial genes (*COI* and *ND5*) to compare the genetic diversity and structure of the two species.

**Results:**

We obtained a 658-bp sequence of *COI* and a 423-bp sequence of *ND5* from both mosquito species. We found low diversity and a nonsignificant population genetic structure in *Ae. albopictus*, and high diversity and a nonsignificant structure in *Ae. flavopictus* for these two mitochondrial genes. *Aedes albopictus* had fewer haplotypes with respect to the number of individuals, and a slight mismatch distribution was confirmed. By contrast, *Ae. flavopictus* had a large number of haplotypes compared with the number of individuals, and a large unimodal-type mismatch distribution was confirmed. Although the genetic structure of both species was nonsignificant, *Ae. flavopictus* exhibited higher genetic diversity than *Ae. albopictus.*

**Conclusions:**

*Aedes albopictus* appears to be an introduced species, whereas *Ae. flavopictus* is endemic to the Korean peninsula, and the difference in genetic diversity between the two species is related to their adaptability and introduction history. Further studies on the genetic structure and diversity of these mosquitos will provide useful data for vector control.

**Supplementary Information:**

The online version contains supplementary material available at 10.1186/s13071-021-04873-5.

## Background

Arthropod-borne viruses are transmitted by blood-sucking insects to animals and humans. Most of them are transmitted by mosquitoes [1, 2]. There are 43 genera and 3583 species of mosquitoes in the world; however, species belonging to the genera *Aedes*, *Anopheles*, and *Culex* are the main vectors of mosquito-borne diseases [3, 4]. In particular, mosquitoes belonging to the genus *Aedes* are the main vectors for spreading fatal diseases such as chikungunya, dengue fever, yellow fever, and Zika virus, that often occur in Asian countries [5–7]. As mosquito-borne diseases may grow in the future due to fast globalization and climate change, information on genetic studies effective in vector monitoring is needed to prevent infectious diseases [2, 8].

Mitochondrial genes are widely used in research on molecular evolution and population genetics of vector insects. Because they have a relatively high mutation rate and high levels of polymorphism and divergence due to their inherent sensitivity, they are highly useful as molecular markers [9–12]. Many vector studies have investigated where the population was introduced using mitochondrial genes [13, 14]. In particular, there have been many studies using *COI* and *ND5* as markers, for example, to determine whether a species has been introduced or to determine the genetic diversity of a population [15, 16]. Population structure and genetic diversity between populations can affect vector capacity [17]. An understanding of these factors is necessary for vector control [18].

*Aedes albopictus*, originally from Southeast Asia, has recently spread throughout all parts of the world except Antarctica, and is considered one of the most dangerous alien species [19–21]. The first record of *Ae. albopictus* in South Korea was in 1940, and its distribution has recently expanded throughout the Korean peninsula [22, 23]. Together with *Aedes aegypti*, substantial research attention has been paid to *Ae. albopictus* as major players in the transmission of vector-borne diseases [24–26]. The main reason for the global spread is that larvae are introduced through used tires, bamboo, etc., due to human activities [27, 28]. Additionally, the range of habitats they can live in has widened as a result of the temperature rise due to global warming [29, 30]. Eggs of *Ae. albopictus* have been shown to tolerate cold weather, and have the potential to expand its distribution in colder regions [31, 32].

*Aedes flavopictus* is known to be native to East Asia, and is divided into three subspecies depending on the region. The subspecies are morphologically and genetically distinct [33, 34]: *Ae. flavopictus*, *Ae. flavopictus downsi*, and *Ae. flavopictus miyarai*. Among the three, *Ae. flavopictus* is distributed in the Korean Peninsula, and records show that they have existed here for a long time, but there have been few molecular studies on this species, so the extent of its genetic diversity is not fully known [33, 35, 36]. *Aedes flavopictus* eggs have been found to survive in colder environments than *Ae. albopictus* eggs [37], and the distribution of eggs has expanded from East Asia to European countries in recent times [38–40]. According to the results of continuous monitoring on the Korean Peninsula, the frequency of appearance of *Ae. flavopictus* is not high [23, 41–43]. *Aedes flavopictus* is not known to act as a vector like *Ae. albopictus* and other *Aedes* species, but it has previously been shown that it may propagate dengue fever [33, 44, 45].

Since the two species are distributed over a wide area in Korea and Japan and share a common habitat [34, 46, 47], research on their overlapping distribution is gradually increasing. Furthermore, there is a possibility of interspecific crossing [48–50]. Not only do the distributions overlap, but the two morphologies are also similar [34, 40, 51], and Japanese studies have shown that the two are phylogenetically close to each other [52, 53]. As *Ae. albopictus* and *Ae. flavopictus* are closely related species and have similar ecological roles and habitats, they can be compared to each other.

The Korean Peninsula has various climates and geographical environments, and the diversity of arthropods that transmit arthropod-borne viruses is also high [54, 55]. There are 11 genera and 56 species of mosquitoes in Korea, including 19 species in the genus *Aedes*. The presence of *Ae. albopictus* and *Ae. flavopictus* was recorded in Korea in the past [22, 35, 56, 57]. Since malaria and Japanese encephalitis occur frequently in Korea, studies have only focused on the vectors of these conditions, and the genus *Aedes* has not been thoroughly investigated [58–60]. There are cases in which foreign mosquitoes have become indigenous, thereby bringing infections from abroad. Additionally, Korea has steadily imported patients, so it is not possible to say that it is a clean country for viruses mediated by *Aedes*; therefore, a preemptive control strategy needs to be established [61, 62].

This study compares the genetic diversity and genetic structure of two species of *Aedes* mosquitoes living in Korea using two mitochondrial genes, with the aim of monitoring mosquito populations. With this work, we intend to create genetic data that infer the genetic state of vectors, to establish a vector control strategy.

## Methods

### Sampling and DNA extraction

**Fig. 1:**
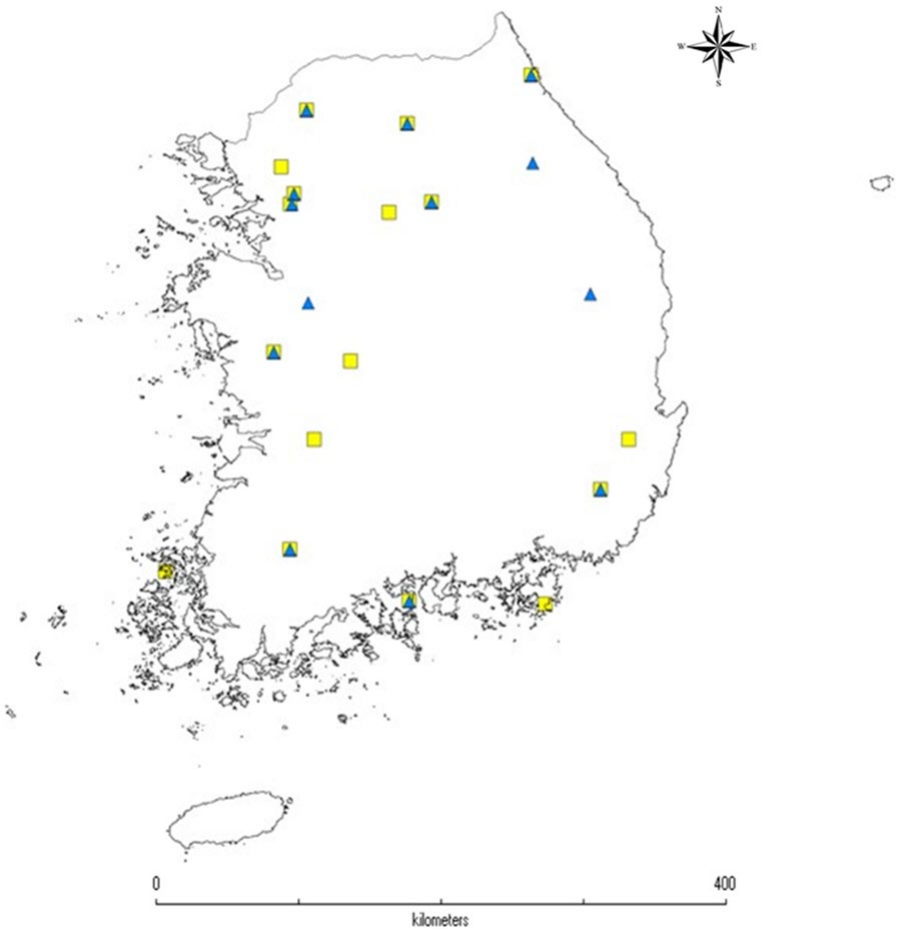
Map of Korea with the sampling locations used in this study. The squares indicate *Aedes albopictus* and the triangles indicate *Aedes flavopictus.* In some populations the two species overlap. DIVA-GIS (version 7.5, www.diva-gis.org) was used to produce a distribution map based on the geographic coordinates of the locality

A total of 314 individual mosquitos were sampled in Korea between 2017 and 2020, including 148 individuals of *Ae. albopictus* from 19 locations and 166 individuals of *Ae. flavopictus* from 14 locations (Fig. 1). All specimens were sampled from the forests, parks and rural areas (Additional file 1: Figures S1–S3). Adult mosquitoes were collected using nets and BG-Sentinel traps (Biogents AG, Germany). All mosquitoes were identified according to the Korean mosquito taxonomic keys [22, 57]. These two species differ in the patch at the root of the front wing. Specimens were individually preserved in tubes filled with 80% ethanol and stored at 4 °C until DNA extraction. DNA was extracted from one to three legs of each sample using the DNeasy Blood & Tissue Kit (Qiagen, Valencia, CA, USA).

### Polymerase chain reaction (PCR) and sequencing

Two regions of mitochondrial genes (*COI* and *ND5*) were amplified by PCR using the following primer pairs: albCOIF (5′-TTTCAACAAATCATAAAGATATTGG-3′) and albCOIR (5′-TAA ACTTCTGGATGACCAAAAAATCA-3′) for COI [63], and ND5_6500F (5′-TCCTTAGAATAAAATCCCGC-3′) and ND5_7398R (5′-GTTTCTGCTTTAGTTCATTCTTC-3′) for ND5 [64]. For COI, PCR amplifications were performed in a 25 μl reaction volume containing 0.5 μl of isolated DNA, 2.5 μl of 10× Taq buffer, 2.0 μl of MgCl_2_ (25 mM), 0.7 μl of dNTP solution (2.5 mM each), 0.5 μl of each primer, and 0.3 μl of Taq DNA polymerase (Takara Bio Inc., Kusatsu, Shiga, Japan). The PCR cycling conditions were as follows: an initial denaturation step at 95 °C for 5 min; followed by 35 cycles of denaturation at 95 °C for 30 s, annealing at 45 °C for 30 s, and elongation at 72 °C for 45 s; with a final extension at 72 °C for 7 min. For ND5, the PCR mixture was the same as that used for COI. The amplification conditions were as follows: initial denaturation at 98 °C for 5 min; followed by 10 cycles at 95 °C for 1 min, 45 °C for 1 min, and 72 °C for 1 min 30 s; 30 cycles at 95 °C for 1 min, 46 °C for 1 min, and 72 °C for 1 min 30 s; and a final extension at 72 °C for 3 min. PCR products were separated by 2% agarose gel electrophoresis (Sigma-Aldrich, Germany) and sequenced by Cosmo Genetech (Seoul, Korea) using the ABI 3730xl DNA Analyzer (Applied Biosystems, Foster City, CA, USA).

### Data analyses

The sequences of the two mitochondrial genes were aligned using the ClustalW plugin on Geneious Prime 2020.1.2 (https://www.geneious.com) and prepared as concatenated sequences. DnaSP 6.12.03 [65] was used for the genetic diversity analysis of mitochondrial DNA, in which the number of haplotypes (*H*), number of segregating sites (*S*), haplotype diversity (Hd), nucleotide diversity (*π*), and average number of nucleotide differences (*k*) were examined.

Pairwise *F*_ST_ values were estimated using Arlequin 3.5 software [66] to investigate genetic differentiation among the populations. Principal coordinate analysis (PCoA) was performed with GenAlEx version 6.51b2 [67] based on pairwise *F*_ST_ values.

Analyses of molecular variance (AMOVA) were performed using Arlequin 3.5 [66] with the locus-by-locus option and using 1000 permutations to determine the population structure. Specimens were grouped according to regional groups in South Korea: Group 1 comprised specimens from Gyeonggi-do, Group 2, Gangwon-do; Group 3, Chungcheong-do; Group 4, Gyeongsang-do, and Group 5, Jeolla-do.

To better understand the genealogical relationships, the haplotypes were constructed using the TCS method as implemented in PopART 1.7 [68].

To investigate the demographic history of populations, deviations from selective neutrality were tested by Tajima’s *D* [69] and Fu’s *F*_S_ [70] metrics using Arlequin 3.5 [66]. To confirm whether a population had undergone sudden expansion, a mismatch distribution was determined using DnaSP 6.12.03 [65].

## Results

Mitochondrial gene sequence analysis resulted in a *CO1* sequence of 658 bp and an *ND5* sequence of 423 bp in the 19 populations (148 individuals) of *Ae. albopictus*, and sequences of the same length were obtained for the 14 populations (166 individuals) of *Ae. flavopictus* (Additional file 2: Table S1). The average winter temperature and average precipitation during the sampling year were obtained based on data from the Korea Meteorological Agency (https://data.kma.go.kr/cmmn/main.do). In the two mitochondrial DNA concatenated sequences, there were 25 haplotypes in *Ae. albopictus* and 107 haplotypes in *Ae. flavopictus*.

The genetic diversity analysis revealed a relatively low number of haplotypes in *Ae. albopictus* compared to the total number of individuals, with relatively low haplotype diversity (0.396) and nucleotide diversity (0.00075) in the entire population. The highest haplotype diversity was found for the 2018 Anyang and Gyeongju populations, and the lowest values were 0 for six populations. The 2018 Anyang and Gyeongju populations also exhibited the highest nucleotide diversity. *Ae. flavopictus* showed a relatively high number of haplotypes compared to the total number of individuals, and high levels of haplotype diversity (0.990) and nucleotide diversity (0.00894) were found in the entire population. Analysis of the two species revealed that *Ae. flavopictus* exhibited higher levels in various genetic diversity indices (Table 1).

**Table 1:** Sampling locations, summary of molecular diversity for each species of this study

Species	Population	Sample size	*H*	*S*	*k*	Hd	*π*	Tajima’s *D*	Fu’s *F*_*S*_
*Aedes albopictus*	2017_Wonju	18	5	4	0.725	0.549	0.00067	−1.12822	−**2.0958**
	2020_Wonju	18	2	3	0.333	0.111	0.00031	−1.71304	0.65061
	Yeoncheon	5	1	0	0	0	0	0	0
	Yangsan	12	6	4	1.152	0.758	0.00107	−0.45947	−**2.89747**
	2020_Anyang	4	2	1	0.500	0.500	0.00046	−0.61237	0.17185
	2018_Anyang	4	4	6	3.167	1.000	0.00293	−0.31446	−1.15708
	Chuncheon	7	1	0	0	0	0	0	0
	Cheongyang	2	1	0	0	0	0	0	0
	Daejeon	8	1	0	0	0	0	0	0
	Gwacheon	7	3	2	0.571	0.524	0.00053	−1.23716	−0.9218
	Geoje	5	2	1	0.600	0.600	0.00056	1.22474	0.62615
	Gwangju	2	1	0	0	0	0	0	0
	Gyeongju	2	2	3	3.000	1.000	0.00278	0	1.09861
	Jeung-do	5	3	2	1.000	0.800	0.00093	0.24314	−0.47542
	Jeonju	28	3	2	0.143	0.140	0.00013	−1.5106	−2.26798
	Sokcho	13	3	2	0.308	0.295	0.00028	−1.46801	−1.40150
	Seoul	3	2	1	0.667	0.667	0.00062	0	0.20067
	Yeoju	3	1	0	0	0	0	0	0
	Yeosu	2	1	0	0	0	0	0	0
*Aedes flavopictus*	2017_Wonju	6	6	19	8.667	1	0.00802	0.25884	−1.18145
	2017_Uiwang	21	16	50	15.281	0.971	0.01414	0.39649	−1.61069
	2020_Uiwang	10	9	29	9.089	0.978	0.00841	−0.54358	−1.86104
	Yeoncheon	2	2	1	1.000	1	0.00093	0	0
	Yangsan	17	14	26	6.75	0.971	0.00624	−0.49372	−5.0955
	Asan	22	15	49	9.779	0.939	0.00905	−1.07423	−2.0798
	Bonghwa	18	16	25	5.693	0.987	0.00527	-0.86027	-9.17073
	Chuncheon	2	2	9	9	1	0.00833	0	2.19722
	Cheongyang	9	8	15	4.944	0.972	0.00457	−0.50238	−2.75282
	Gwacheon	10	9	26	10.133	0.978	0.00937	0.48927	−1.59674
	Gwangju	17	8	33	7.721	0.816	0.00714	−0.85617	1.62731
	Pyeongchang	9	7	27	8.861	0.917	0.00820	−0.53942	0.02987
	Sokcho	6	5	14	5.333	0.933	0.00493	−0.79924	−0.2382
	Yeosu	17	6	20	5.412	0.691	0.00501	−0.33724	2.44814

Bold cases represent significance at *P* < 0.05

**Fig. 2:**
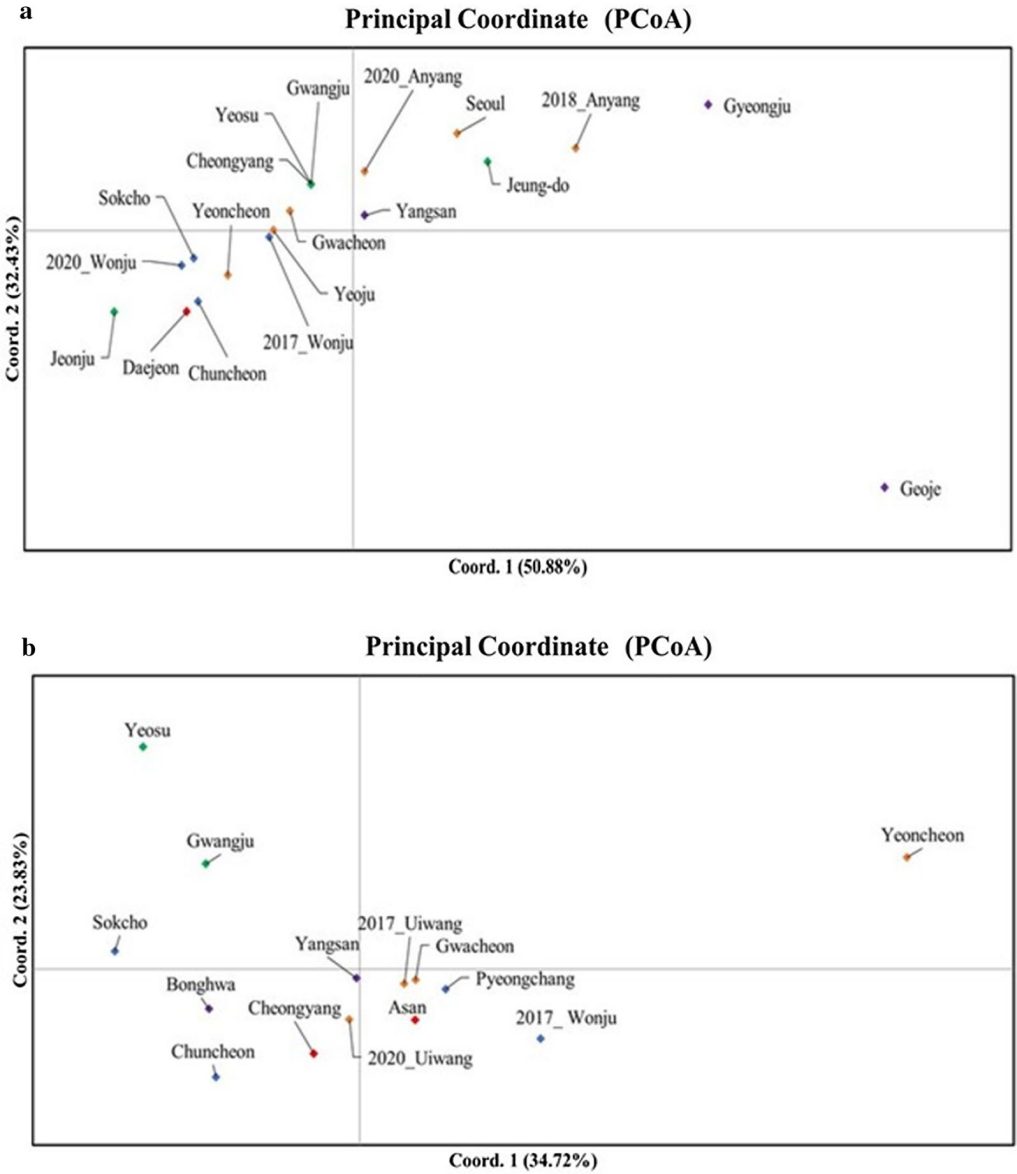
Principal coordinate analysis (PCoA) plot of pairwise population *F*_ST_ values for the locations sampled in Korea in this study. **a** *Aedes albopictus*; **b** *Aedes flavopictus*

*Aedes albopictus* showed nonsignificant pairwise *F*_ST_ values overall, but the Geoje population showed a high level of significance in structure compared to the other populations. By contrast, *Ae. flavopictus* showed low overall *F*_ST_ values, among which those of the Yeosu and Yeoncheon populations were significant (Fig. 2; Additional file 3: Tables S2, S3). In both species, there were no significant pairwise *F*_ST_ values among domestic populations except for a few populations.

AMOVA showed low genetic variance among both species, but high variance within populations. In particular, *Ae. flavopictus* showed higher variance than *Ae. albopictus*, indicating that it can form a genetic structure within populations (Table 2).

**Table 2:** Analysis of molecular variance (AMOVA) of this study

Species	Source of variation	Degrees of freedom	Percentage of variation (%)
*Aedes albopictus*	Among groups	4	−5.6
	Among populations within groups	14	46.4
	Within populations	129	59.2
*Aedes flavopictus*	Among groups	4	4.0
	Among populations within groups	9	9.6
	Within populations	152	86.3

**Fig. 3:**
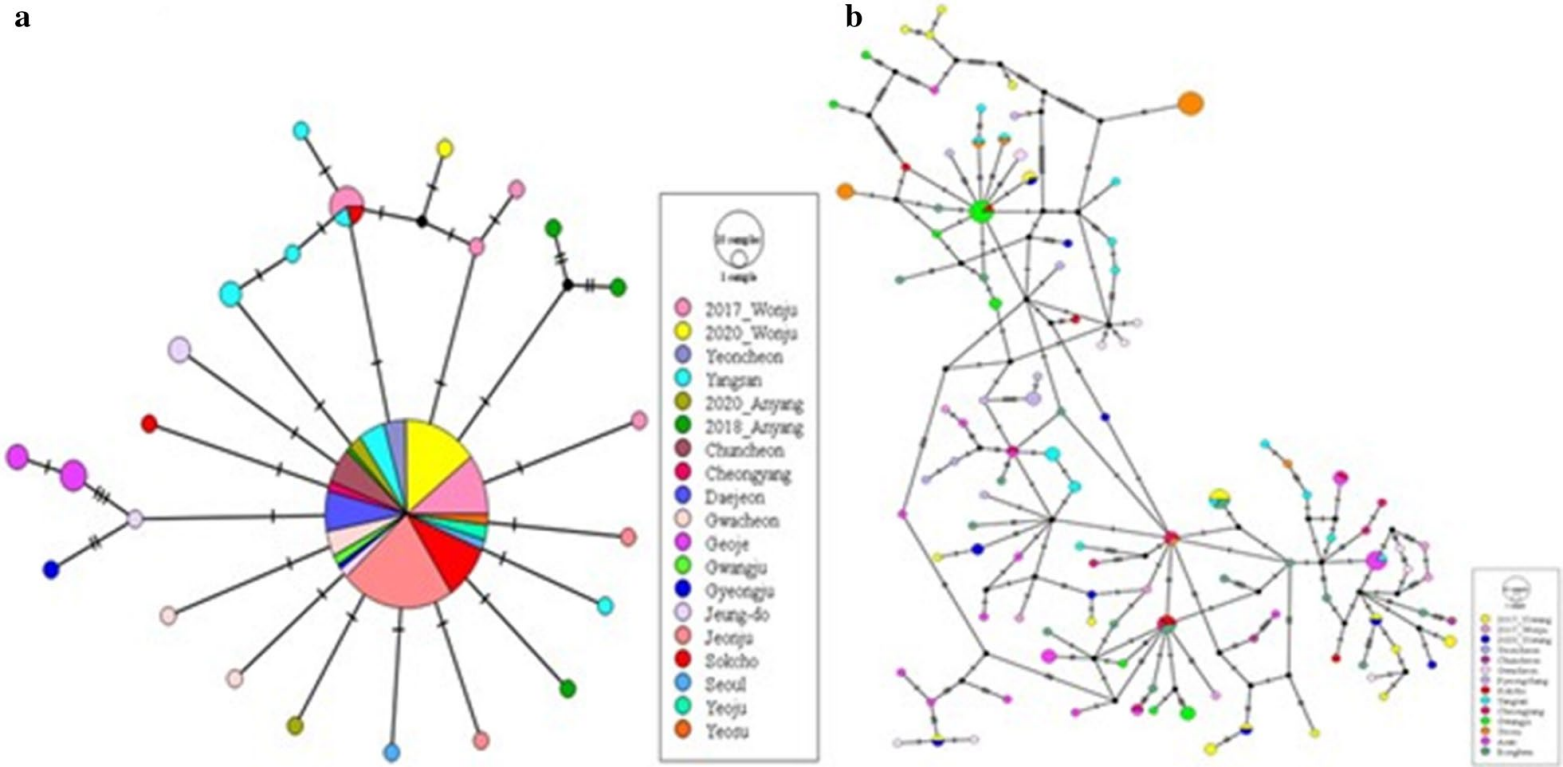
TCS networks constructed with PopART for haplotypes. **a** *Aedes albopictus*; **b** *Aedes flavopictus.* Circle sizes reflect haplotype abundance and percentage of color in the circles shows the haplotype frequency

In the haplotype network, *Ae. albopictus* showed a simple star-like form, in which several haplotypes diverged from one of the largest haplotypes, and hap_1 had the highest frequency of 78% in all populations. Private haplotypes, most of which were singleton haplotypes, accounted for 22%. *Aedes flavopictus* exhibited a complex haplotype network, which was found to have a higher haplotype frequency compared to the total number of individuals. Most of the network was composed of singleton haplotypes. When comparing the two species, *Ae. flavopictus* had more haplotypes than *Ae. albopictus* and showed a complex haplotype network (Fig. 3).

**Fig. 4:**
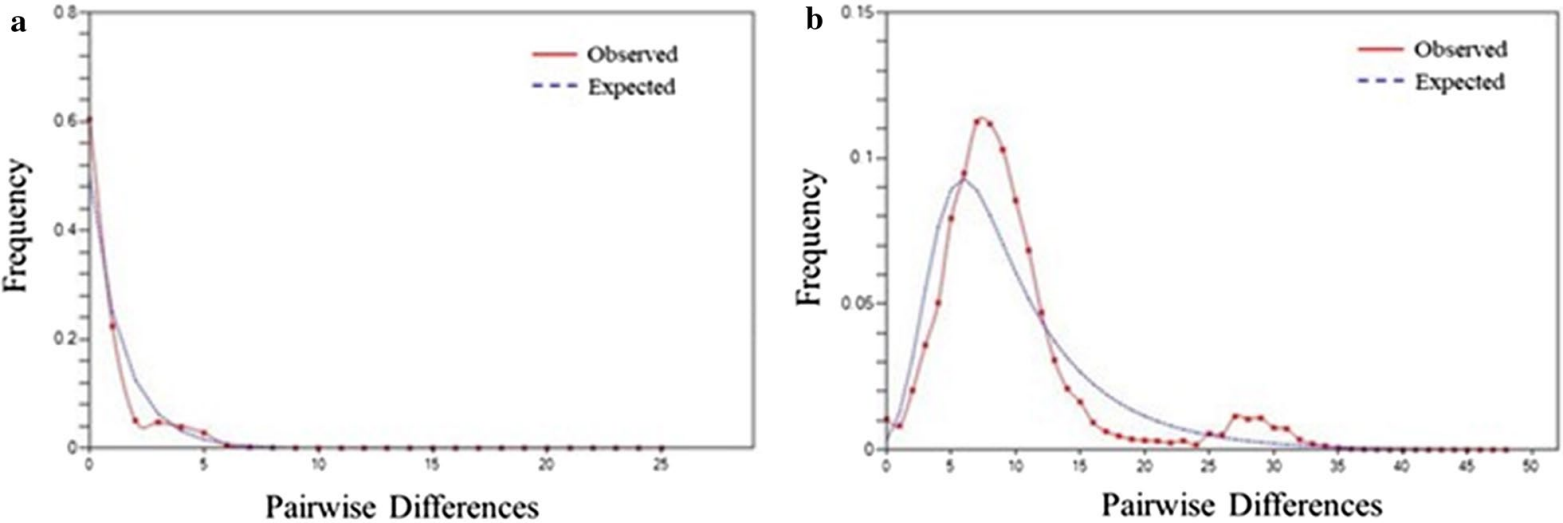
Mismatch distribution of two species populations in Korea. **a** *Aedes albopictus*; **b** *Aedes flavopictus*

With respect to demographic history, *Ae. albopictus* showed negative but low values for Tajima’s *D* (−0.36713) and Fu’s *F*_*S*_ (−0.44574) in the whole population. Negative values of Tajima’s *D* (−0.34726) and Fu’s *F*_*S*_ (−1.37746) were also found for the entire population of *Ae. flavopictus*. In both species, *Ae. flavopictus* showed negative Fu’s *F*_*S*_ values. For the mismatch distribution, the result of *Ae. albopictus* was nonsignificant, whereas *Ae. flavopictus* showed a large unimodal shape, indicating the possibility of sudden expansion of the population (Fig. 4).

## Discussion

Based on the above results, two conclusions can be drawn. Higher genetic diversity was observed in *Ae. flavopictus* than in *Ae. albopictus*, and the two species of mosquitoes generally showed low levels of genetic structure except for some populations.

There are two hypotheses addressing the overall low diversity of *Ae. albopictus* in Korea. The first concerns the spread of *Wolbachia* to the mitochondria: in many arthropods, selective sweeps arising from widespread *Wolbachia* infections often lead to low diversity, therefore posing a high possibility of unreliable results [71]. Previous studies have shown that intracellular *Wolbachia* was detected in 17 populations in Korea, and these groups are known to have low mitochondrial diversity [72, 73]. However, there is no clear evidence of *Wolbachia* in many samples, and further analysis of the nuclear genes, as well as the mitochondrial genes, will be needed to reveal the impact of *Wolbachia* on genetic diversity in the population [72, 74]. The second hypothesis is that the introduced *Ae. albopictus* may have been affected by Korea’s harsh winter climate, which is different from that of the country of origin. *Ae. albopictus* is considered to be an invasive species that has recently spread abroad from its origin in Southeast Asia [19, 20]. These mosquitos have adapted to the environment of each country since their introduction, but previous studies have shown that they also have low diversity in the countries from which they were introduced [13, 15]. The environment in Southeast Asia, the native habitat of *Ae. albopictus*, is hot and humid, facilitating the spread of *Ae. albopictus* [75]. However, winters are quite severe in Korea, with cold, dry weather. The average temperature is less than 10 °C. This environment could lead to a decrease in the population size of this mosquito, resulting in decreased genetic diversity [60, 76]. In the domestic populations, the Geoje population is different from other populations. This is believed to arise from genetic differences due to the physical distance between mosquitoes introduced by human activities. The distance traveled by mosquitoes in the natural environment is, however, only a few kilometers [77], and there are study results showing that the populations have been genetically structured in heterogeneous habitats due to their limited dispersive abilities [78].

Korean *Ae. flavopictus* have high genetic diversity and a complex haplotype network. There are two hypotheses regarding their high diversity. The first is based on the fact that *Ae. flavopictus* is endemic to East Asia. Studies on mosquitoes of the genus *Aedes* show that genetic diversity in the original population is much higher, which supports the contention that the original population of *Ae. flavopictus* is Korean [79, 80]. The second hypothesis considers the adaptation to cold climates as an endemic species: *Ae. flavopictus* is an Asian species that does not exist in tropical regions, and lives in subtropical areas throughout the cool-temperate region [53]. This mosquito species has recently been found in the Netherlands, a more northerly region, and is considered highly likely to spread due to its ability to cope with environmental changes [39, 40]. *Aedes flavopictus* has excellent environmental adaptability, and its eggs can survive in cold and dry conditions for long periods [35, 81]. Studies have also shown that it is genetically close to *Aedes galloisi*, a northern mosquito species in the same genus [52].

Differences in diversity between *Ae. albopictus* and *Ae. flavopictus* can be explained in several ways. A small mismatch in distribution and a single haplotype shared by various populations indicate that the patterns observed in *Ae. albopictus* may have been affected by a decrease in effective population size, human introduction, and natural environmental changes [13, 15, 82, 83]. Environmental and biological barriers, and factors such as human activity, climate change, migration, and genetic flow can affect the genetic diversity and structure of species [84]. *Aedes flavopictus*, an endemic species, shows high genetic diversity, a large unimodal mismatch distribution, and a complex haplotype network. However, the unimodal form of its mismatch distribution indicates that the *Ae. flavopictus* population may have recently experienced a large population expansion. This successful distribution and increasing population of the endemic *Ae. flavopictus* may have been affected by human demographics [25]; further, the complex form of the haplotype network indicates a high mutation rate, which can increase the rate of resistance development in insects [85, 86]. The difference in genetic diversity between these two mosquito species living in Korea may also arise from the differences in effective population size due to their ability to adapt to the cold as well as their status as an endemic or introduced species. Although the distribution of these two species overlaps, *Ae. albopictus* can survive for up to 24 h at −10 °C in the form of diapause eggs [87], and the eggs of *Ae. flavopictus* can survive for a longer period [35, 37, 53]. The decreased survival rate of eggs can affect the effective population size, as fewer adults develop [88, 89]. This difference in cold adaptation and consequent effects on the size of the effective population can lead to differences in genetic diversity [60, 90, 91]. Monitoring of vectors in Korea has shown that the frequency of *Ae. flavopictus* appearance was not high, but the reason for the large potential population size in this study lies in the difference between the location and the collection method [23, 41–43]. Thus, continuous monitoring is needed because this species is highly likely to affect humans, as it has a large population size and considerable potential as a vector.

The differences in the genetic diversity of *Ae. albopictus* and *Ae. flavopictus* populations revealed in this study suggest that the continuous monitoring of these species with multiple possibilities as vectors is essential. To understand the genetic diversity of *Aedes* mosquito species in Korea, sampling in more diverse regions and the use of different genetic markers will be conducted in further studies.

## Conclusions

This is the first paper comparing genetic diversity and the genetic structure of two *Aedes* mosquito species inhabiting Korea. The results showed that *Ae. albopictus*, which is considered to be an introduced species, has lower genetic diversity than *Ae. flavopictus*, the endemic species. The low diversity of *Ae. albopictus* suggests that these mosquitos were introduced by humans, but did not fully adapt to the environment of the Korean Peninsula. The high diversity of *Ae. flavopictus* could be due to its greater adaptability to the environment of the Korean Peninsula as an endemic species, but may also be influenced by an increase in population and resistance to pesticides. However, in light of the rising temperatures caused by climate change, the domestic inflow of patients, and the population density, the Korean Peninsula will continue to face the threat of mosquito-borne diseases. Studies of the genetic status of potential vector species will provide useful data for inferring effective population sizes and monitoring and managing mosquito populations.

## Supplementary Information

**Additional file 1: Figure S1.** Representative picture of a forest area surveyed in this study in Korea. **Figure S2.** Representative picture of a rural area surveyed in this study in Korea. **Figure S3.** Representative picture of a park surveyed in this study in Korea.

**Additional file 2: Table S1.** Details of the mosquito populations used in this study, with GenBank accession numbers for *CO1* and *ND5* gene sequences.

**Additional file 3: Table S2.** Pairwise *F*_ST_ values obtained using two mitochondrial DNA concatenated sequences from *Aedes albopictus*. **Table S3.** Pairwise *F*_ST_ values obtained using two mitochondrial DNA concatenated sequences from *Aedes flavopictus*.

## Data Availability

Accession numbers for mitochondrial DNA sequences generated in this study are in Additional file 2: Table S1.

## Abbreviations

COI: Cytochrome c oxidase subunit I
ND5: NADH dehydrogenase subunit 5
