# Supplementary material for: Comparative population genetics of the invasive mosquito Aedes albopictus and the native mosquito Aedes flavopictus in the Korean peninsula
Source: Parasit Vectors. 2021 Jul 27;14:377. doi: 10.1186/s13071-021-04873-5 (PMC8314453; doi:10.1186/s13071-021-04873-5)
Supplement: Supplementary file 1 — Additional file 1: Figure S1. Representative picture of a forest area surveyed in this study in Korea. Figure S2. Representative picture of a rural area surveyed in this study in Korea. Figure S3. Representative picture of a park surveyed in this study in Korea. [file 13071_2021_4873_MOESM1_ESM.docx]

**
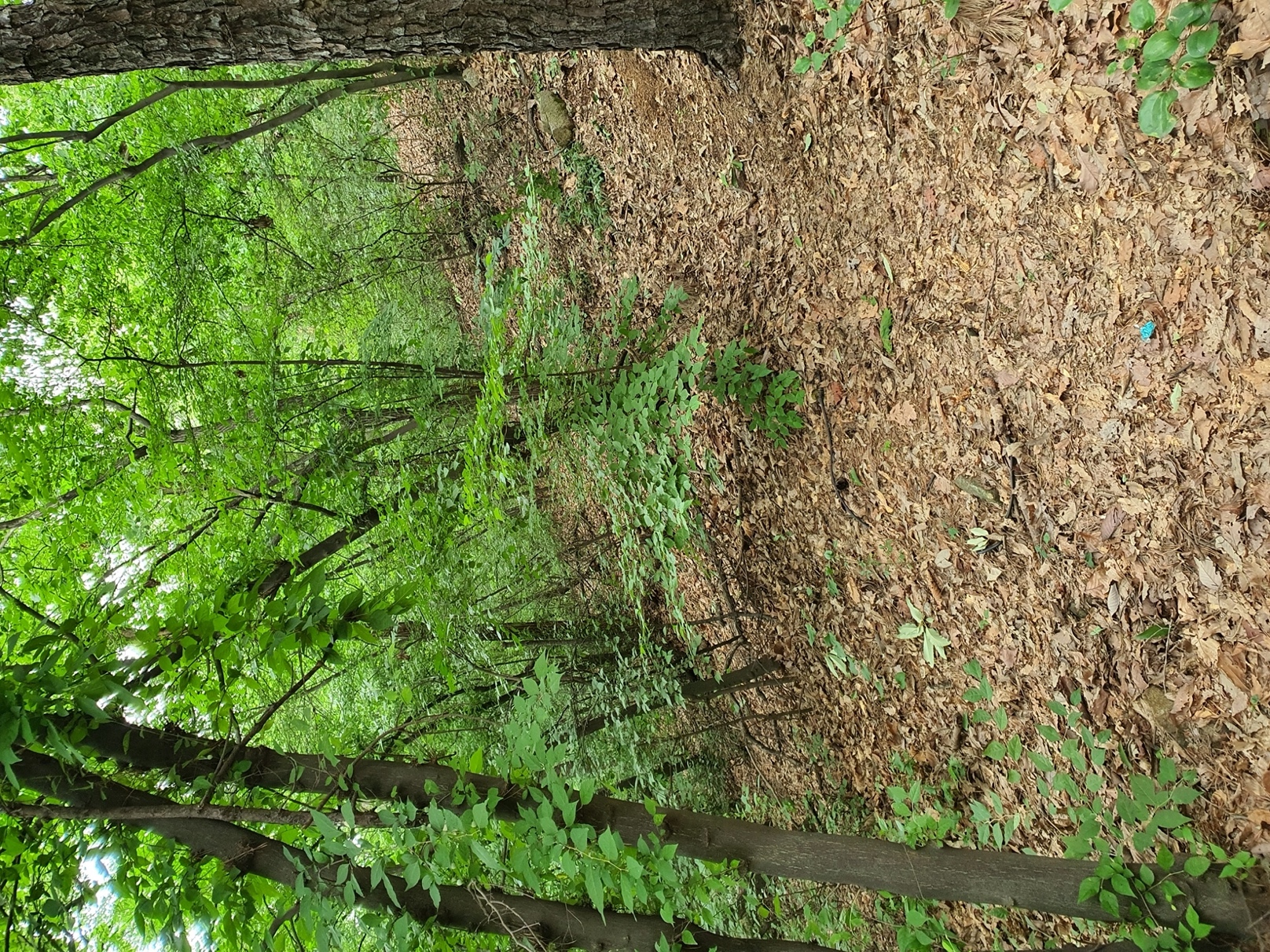
**

**Additional file 1: Figure S1.** Representative picture of a forest area surveyed in this study in Korea.

**
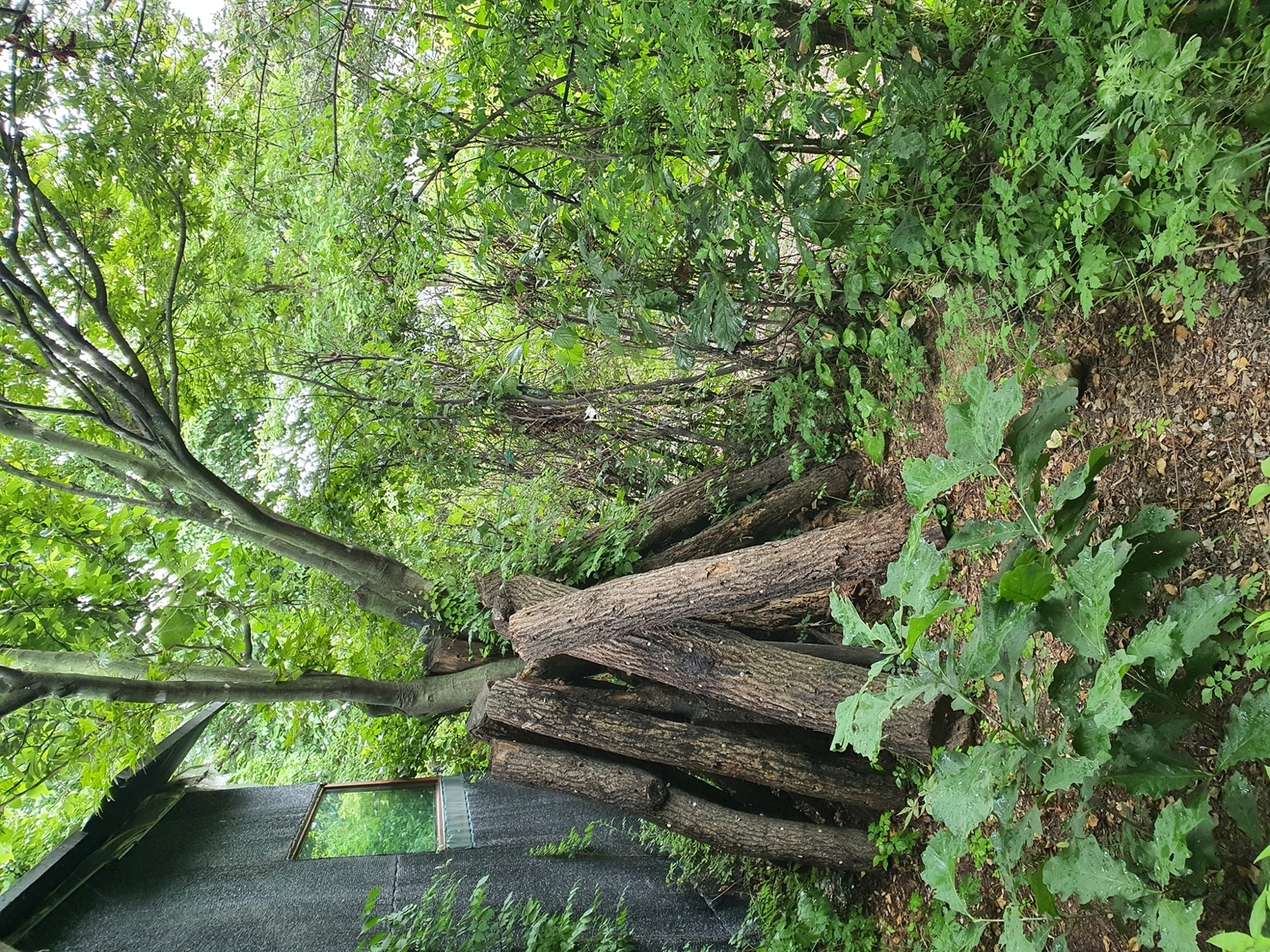
**

**Additional file 1: Figure S2.** Representative picture of a rural area surveyed in this study in Korea.

**
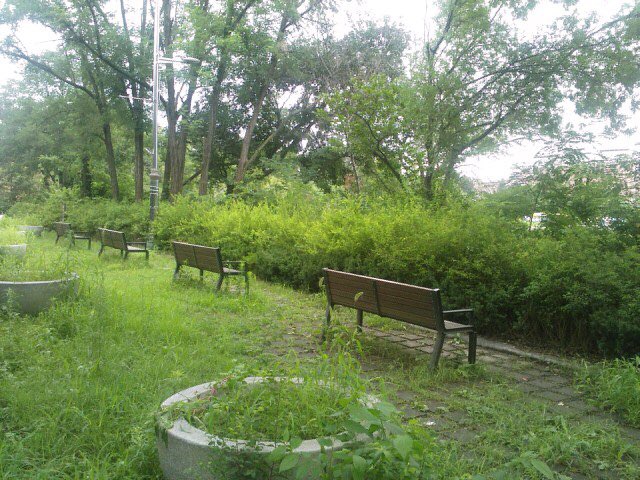
**

**Additional file 1: Figure S3.** Representative picture of a park surveyed in this study in Korea.
