# Supplementary material for: Comparative population genetics of the invasive mosquito Aedes albopictus and the native mosquito Aedes flavopictus in the Korean peninsula
Source: Parasit Vectors. 2021 Jul 27;14:377. doi: 10.1186/s13071-021-04873-5 (PMC8314453; doi:10.1186/s13071-021-04873-5)
Supplement: Supplementary file 2 — Additional file 2: Table S1. Details of the mosquito populations used in this study, with GenBank accession numbers for CO1 and ND5 gene sequences. [file 13071_2021_4873_MOESM2_ESM.docx]

**Additional file 2: Table S1**. Details of the mosquito populations used in this study, with GenBank accession numbers for *CO1* and *ND5* gene sequences.

| Species | Population | Latitude, longitude | Collection year | Habitat | Average winter temperature | Average precipitation | GenBank accession numbers | |
| --- | --- | --- | --- | --- | --- | --- | --- | --- |
|  |  |  |  |  |  |  | *CO1* | *ND5* |
| *Aedes albopictus* | 2017_Wonju | 37°22'52.6"N 127°53'36.1"E | 2017.08 | Rural | -2.5℃ | 1063.6 | MW526509-MW526526 | MW526720-MW526737 |
|  | 2020_Wonju | 37°22'52.6"N 127°53'36.1"E | 2020.07 | Rural | -0.6℃ | 1317 | MW526527-MW526544 | MW526738-MW526755 |
|  | Yeoncheon | 37°58'35.6"N 127°04'05.3"E | 2017.07 | Forest | -3.8℃ | 989.7 | MW526545-MW526549 | MW526756-MW526760 |
|  | Yangsan | 35°31'13.9"N 129°00'40.1"E | 2017.07 | Forest | 2℃ | 720.2 | MW526550-MW526561 | MW526761-MW526772 |
|  | 2020_Anyang | 37°22'01.7"N 126°57'39.0"E | 2020.08 | Park | -0.2℃ | 1635.5 | MW526562-MW526565 | MW526773-MW526776 |
|  | 2018_Anyang | 37°22'01.7"N 126°57'39.0"E | 2018.06 | Park | -0.2℃ | 1293.1 | MW526566-MW526569 | MW526777-MW526780 |
|  | Chuncheon | 37°53'15.0"N 127°44'02.9"E | 2017.08 | Forest | -4.1℃ | 1217.1 | MW526570-MW526576 | MW526781-MW526787 |
|  | Cheongyang | 36°24'28.5"N 126°51'04.8"E | 2017.09 | Forest | -0.5℃ | 856.2 | MW526577-MW526578 | MW526788-MW526789 |
|  | Daejeon | 36°21'02.3"N 127°21'35.5"E | 2017.09 | Forest | -1.1℃ | 1127.5 | MW526579-MW526586 | MW526790-MW526797 |
|  | Gwacheon | 37°26'00.0"N 126°59'00.1"E | 2020.07 | Forest | -0.2℃ | 1635.5 | MW526587-MW526593 | MW526798-MW526804 |
|  | Geoje | 34°46'43.8"N 128°38'42.1"E | 2017.07 | Forest | 2.8℃ | 1411.1 | MW526594-MW526598 | MW526805-MW526809 |
|  | Gwangju | 35°07'53.0"N 126°57'30.0"E | 2017.09 | Forest | 1.2℃ | 936.6 | MW526599-MW526600 | MW526810-MW526811 |
|  | Gyeongju | 35°50'41.4"N 129°11'43.4"E | 2017.07 | Forest | 0.2℃ | 590.7 | MW526601-MW526602 | MW526812-MW526813 |
|  | Jeung-do | 34°59'15.8"N 126°08'07.3"E | 2017.07 | Forest | 1.3 | 722.5 | MW526603-MW526607 | MW526814-MW526818 |
|  | Jeonju | 35°50'37.2"N 127°07'09.0"E | 2017.07 | Park | 0℃ | 947.8 | MW526608-MW526635 | MW526819-MW526846 |
|  | Sokcho | 38°12'10.5"N 128°33'07.5"E | 2020.09 | Forest | 1.2℃ | 2085.8 | MW526636-MW526648 | MW526847-MW526859 |
|  | Seoul | 37°36'32.9"N 126°54'06.5"E | 2020.07 | Forest | 0℃ | 1651.1 | MW526649-MW526651 | MW526860-MW526862 |
|  | Yeoju | 37°18'43.5"N 127°36'52.8"E | 2020.07 | Forest | -1℃ | 1668.8 | MW526652-MW526654 | MW526863-MW526865 |
|  | Yeosu | 34°47'57.0"N 127°44'52.4"E | 2020.06 | Forest | 4.5℃ | 1751.9 | MW526655-MW526656 | MW526866-MW526867 |
| *Aedes flavopictus* | 2017_Wonju | 37°22'52.6"N 127°53'36.1"E | 2017.08 | Rural | -2.5℃ | 1063.6 | MW539251-MW539256 | MW539085-MW539090 |
|  | 2017_Uiwang | 37°21'57.9"N 126°58'20.9"E | 2017.09 | Forest | -2.2℃ | 1328.6 | MW539230-MW539250 | MW539064-MW539084 |
|  | 2020_Uiwang | 37°21'57.9"N 126°58'20.9"E | 2020.07 | Forest | -0.2℃ | 1635.5 | MW539257-MW539266 | MW539091-MW539100 |
|  | Yeoncheon | 37°58'35.6"N 127°04'05.3"E | 2017.07 | Forest | -3.8℃ | 989.7 | MW539267-MW539268 | MW539101-MW539102 |
|  | Yangsan | 35°31'13.9"N 129°00'40.1"E | 2017.07 | Forest | 2℃ | 720.2 | MW539269-MW539285 | MW539103-MW539119 |
|  | Asan | 36°43'44.6"N 127°04'41.1"E | 2017.09 | Forest | -2.7℃ | 1305 | MW539286-MW539307 | MW539120-MW539141 |
|  | Bonghwa | 36°47'05.0"N 128°56'32.6"E | 2017.08 | Forest | -4.2℃ | 795.3 | MW539308-MW539325 | MW539142-MW539159 |
|  | Chuncheon | 37°53'15.0"N 127°44'02.9"E | 2017.08 | Forest | -4.1℃ | 1217.1 | MW539326-MW539327 | MW539160-MW539161 |
|  | Cheongyang | 36°24'28.5"N 126°51'04.8"E | 2017.09 | Forest | -0.5℃ | 856.2 | MW539328-MW539336 | MW539162-MW539170 |
|  | Gwacheon | 37°26'00.0"N 126°59'00.1"E | 2020.07 | Forest | -0.2℃ | 1635.5 | MW539337-MW539346 | MW539171-MW539180 |
|  | Gwangju | 35°07'53.0"N 126°57'30.0"E | 2017.09 | Forest | 1.2℃ | 936.6 | MW539347-MW539363 | MW539181-MW539197 |
|  | Pyeongchang | 37°38'05.1"N 128°33'48.5"E | 2020.08 | Forest | -2.1℃ | 1305.4 | MW539364-MW539372 | MW539198-MW539206 |
|  | Sokcho | 38°12'10.5"N 128°33'07.5"E | 2020.09 | Forest | 1.2℃ | 2085.8 | MW539373-MW539378 | MW539207-MW539212 |
|  | Yeosu | 34°47'57.0"N 127°44'52.4"E | 2020.06 | Forest | 4.5℃ | 1751.9 | MW539379-MW539395 | MW539213-MW539229 |
