# Supplementary material for: Comparative population genetics of the invasive mosquito Aedes albopictus and the native mosquito Aedes flavopictus in the Korean peninsula
Source: Parasit Vectors. 2021 Jul 27;14:377. doi: 10.1186/s13071-021-04873-5 (PMC8314453; doi:10.1186/s13071-021-04873-5)
Supplement: Supplementary file 3 — Additional file 3: Table S2. Pairwise FST values obtained using two mitochondrial DNA concatenated sequences from Aedes albopictus. Table S3. Pairwise FST values obtained using two mitochondrial DNA concatenated sequences from Aedes flavopictus. [file 13071_2021_4873_MOESM3_ESM.docx]

**Additional file 3: Table S2.** Pairwise *F*_ST_ values obtained using two mitochondrial DNA concatenated sequences from *Aedes albopictus*.

|  | 2017_Wonju | 2020_Wonju | Yeoncheon | 2020_Anyang | 2018_Anyang | Chuncheon | Gwacheon | Sokcho | Seoul | Yeoju | Yangsan | Cheongyang | Daejeon | Geoje | Gwangju | Gyeongju | Jeung-do | Jeonju | Yeosu |
| --- | --- | --- | --- | --- | --- | --- | --- | --- | --- | --- | --- | --- | --- | --- | --- | --- | --- | --- | --- |
| 2017_Wonju | - |  |  |  |  |  |  |  |  |  |  |  |  |  |  |  |  |  |  |
| 2020_Wonju | **-0.009** | - |  |  |  |  |  |  |  |  |  |  |  |  |  |  |  |  |  |
| Yeoncheon | -0.051 | -0.103 | - |  |  |  |  |  |  |  |  |  |  |  |  |  |  |  |  |
| 2020_Anyang | 0.005 | 0.045 | 0.063 | - |  |  |  |  |  |  |  |  |  |  |  |  |  |  |  |
| 2018_Anyang | **0.288** | **0.375** | **0.159** | 0.083 | - |  |  |  |  |  |  |  |  |  |  |  |  |  |  |
| Chuncheon | -0.013 | -0.066 | 0.000 | 0.152 | **0.252** | - |  |  |  |  |  |  |  |  |  |  |  |  |  |
| Gwacheon | 0.027 | 0.027 | -0.055 | -0.008 | 0.183 | 0.000 | - |  |  |  |  |  |  |  |  |  |  |  |  |
| Sokcho | -0.008 | **-0.028** | -0.096 | 0.049 | **0.329** | **-0.055** | **0.023** | - |  |  |  |  |  |  |  |  |  |  |  |
| Seoul | 0.024 | 0.117 | 0.189 | 0.014 | 0.019 | 0.300 | 0.017 | 0.121 | - |  |  |  |  |  |  |  |  |  |  |
| Yeoju | -0.140 | -0.195 | 0.000 | -0.091 | 0.004 | 0.000 | -0.167 | -0.191 | 0.000 | - |  |  |  |  |  |  |  |  |  |
| Yangsan | **0.043** | **0.103** | 0.022 | 0.031 | **0.216** | 0.068 | 0.071 | **0.072** | 0.023 | -0.070 | - |  |  |  |  |  |  |  |  |
| Cheongyang | -0.266 | -0.330 | 0.000 | -0.263 | -0.160 | 0.000 | -0.313 | -0.328 | -0.200 | 0.000 | -0.191 | - |  |  |  |  |  |  |  |
| Daejeon | 0.000 | -0.054 | 0.000 | 0.186 | **0.288** | 0.000 | 0.020 | -0.042 | 0.342 | 0.000 | 0.085 | 0.000 | - |  |  |  |  |  |  |
| Geoje | **0.854** | **0.915** | **0.932** | **0.880** | **0.719** | **0.945** | **0.876** | **0.915** | **0.868** | **0.910** | **0.805** | **0.893** | **0.950** | - |  |  |  |  |  |
| Gwangju | -0.266 | -0.330 | 0.000 | -0.263 | -0.160 | 0.000 | -0.313 | -0.328 | -0.200 | 0.000 | -0.191 | **0.000** | 0.000 | 0.893 | - |  |  |  |  |
| Gyeongju | 0.397 | 0.565 | 0.474 | 0.258 | 0.043 | 0.588 | 0.351 | 0.544 | 0.152 | 0.250 | 0.280 | 0.000 | 0.628 | **0.766** | 0.000 | - |  |  |  |
| Jeung-do | **0.159** | 0.246 | 0.167 | 0.098 | 0.146 | **0.247** | 0.133 | **0.232** | 0.077 | 0.032 | **0.140** | -0.111 | 0.279 | **0.826** | -0.111 | 0.175 | - |  |  |
| Jeonju | **0.080** | 0.009 | -0.108 | 0.181 | **0.530** | -0.072 | 0.097 | **0.018** | 0.311 | -0.198 | **0.210** | -0.332 | -0.061 | **0.954** | -0.332 | 0.737 | **0.405** | - |  |
| Yeosu | -0.266 | -0.330 | 0.000 | -0.263 | -0.160 | 0.000 | -0.313 | -0.328 | -0.200 | 0.000 | -0.191 | 0.000 | 0.000 | 0.893 | 0.000 | 0.000 | -0.111 | -0.332 | - |

Bold cases represent significant at *P*<0.05

**Additional file 3: Table S3.** Pairwise *F*_ST_ values obtained using two mitochondrial DNA concatenated sequences from *Aedes flavopictus*.

|  | 2017_Uiwang | 2017_Wonju | 2020_Uiwang | Yeoncheon | Chuncheon | Gwacheon | Pyeongchang | Sokcho | Yangsan | Asan | Bonghwa | Cheongyang | Gwangju | Yeosu |
| --- | --- | --- | --- | --- | --- | --- | --- | --- | --- | --- | --- | --- | --- | --- |
| 2017_Uiwang | - |  |  |  |  |  |  |  |  |  |  |  |  |  |
| 2017_Wonju | 0.078 | - |  |  |  |  |  |  |  |  |  |  |  |  |
| 2020_Uiwang | 0.020 | 0.059 | - |  |  |  |  |  |  |  |  |  |  |  |
| Yeoncheon | **0.178** | 0.170 | **0.244** | - |  |  |  |  |  |  |  |  |  |  |
| Chuncheon | -0.128 | 0.044 | -0.079 | 0.545 | - |  |  |  |  |  |  |  |  |  |
| Gwacheon | **0.074** | 0.090 | 0.050 | 0.265 | -0.012 | - |  |  |  |  |  |  |  |  |
| Pyeongchang | **0.113** | **0.156** | **0.083** | 0.264 | 0.074 | **0.130** | - |  |  |  |  |  |  |  |
| Sokcho | 0.066 | **0.160** | 0.015 | **0.484** | 0.084 | **0.139** | **0.156** | - |  |  |  |  |  |  |
| Yangsan | **0.122** | **0.098** | 0.032 | **0.282** | 0.114 | **0.106** | **0.118** | 0.064 | - |  |  |  |  |  |
| Asan | **0.088** | 0.061 | 0.024 | 0.215 | -0.071 | **0.056** | **0.070** | **0.091** | **0.081** | - |  |  |  |  |
| Bonghwa | **0.101** | **0.117** | 0.031 | **0.399** | 0.054 | **0.166** | **0.165** | -0.023 | **0.053** | **0.077** | - |  |  |  |
| Cheongyang | **0.106** | 0.079 | 0.041 | **0.409** | 0.166 | **0.169** | **0.141** | 0.096 | 0.039 | **0.058** | 0.010 | - |  |  |
| Gwangju | **0.130** | **0.228** | **0.111** | **0.359** | 0.110 | **0.162** | **0.187** | 0.003 | **0.127** | **0.156** | **0.108** | **0.183** | - |  |
| Yeosu | **0.227** | **0.373** | **0.249** | **0.508** | 0.267 | **0.250** | **0.319** | **0.169** | **0.258** | **0.262** | **0.252** | **0.348** | **0.157** | - |

Bold cases represent significant at *P*<0.05
